# Different European Perspectives on the Treatment of Clinical Mastitis in Lactation

**DOI:** 10.3390/antibiotics11081107

**Published:** 2022-08-16

**Authors:** Franziska Preine, Demetrio Herrera, Christian Scherpenzeel, Piret Kalmus, Finola McCoy, Sebastian Smulski, Päivi Rajala-Schultz, Anne Schmenger, Paolo Moroni, Volker Krömker

**Affiliations:** 1 Department of Bioprocess Engineering, Microbiology, Faculty II, Hannover University of Applied Sciences and Arts, 30453 Hannover, Germany; 2 Veterinary Services Company Q-Llet SLP, 08553 Barcelona, Spain; 3 GD Animal Health, P.O. Box 9, 7400 AA Deventer, The Netherlands; 4 Institute of Veterinary Medicine and Animal Science, Estonian University of Life Sciences, 51006 Tartu, Estonia; 5 Animal Health Ireland, Carrick on Shannon, N41 WN27 County Leitrim, Ireland; 6 Department of Internal Diseases and Diagnostics, Poznan University of Life Sciences, 60637 Poznan, Poland; 7 Department of Production Animal Medicine, Faculty of Veterinary Medicine, University of Helsinki, 04920 Helsinki, Finland; 8 Dipartimento di Medicina Veterinaria e Scienze Animali, Università degli Studi di Milano, 26900 Lodi, Italy; 9 Quality Milk Production Services, Animal Health Diagnostic Center, Cornell University, Ithaca, NY 14853, USA; 10 Department of Veterinary and Animal Sciences, Faculty of Health and Medical Sciences, Section for Production, Nutrition and Health, University of Copenhagen, 1870 Frederiksberg, Denmark

**Keywords:** clinical bovine mastitis, treatment approaches, lactational treatment, Europe, ENOVAT

## Abstract

As part of the European Network for Optimization of Veterinary Antimicrobial Treatment (ENOVAT), a webinar on the topic “Mastitis Treatment in Lactation” was held, in which eight mastitis experts from different European countries (Spain, The Netherlands, Estonia, Ireland, Poland, Finland, Germany, and Italy) presented their treatment approaches for clinical mastitis in lactation. The aim of this study was to compare the therapeutic approaches to identify commonalities and differences. In all eight participating countries, the decision to start treatment is usually made by the veterinarians, while the farm personnel are responsible for treatment administration. Antibiotic treatment is then typically administered intramammarily. The treatment duration often depends on the label instructions and is frequently extended if *Staphylococcus aureus* or *Streptococcus uberis* is involved. Administering supportive therapy, especially non-steroidal anti-inflammatory drugs (NSAIDs) is an established practice in all countries. Penicillin is the first-choice drug for the treatment of mastitis in an increasing number of countries. The use of critically important antimicrobials (CIAs) such as quinolones and third- and fourth-generation cephalosporins is at a low level in Finland and The Netherlands. In Estonia, Germany, Italy, and Spain, the use of CIAs is declining and is only allowed if milk samples are analyzed in advance following the legal framework. Systems for monitoring antibiotic use are being introduced in more and more countries. This exchange of different views will help the European countries to move towards a common high standard of antimicrobial stewardship in veterinary medicine.

## 1. Introduction

The development of antimicrobial resistance (AMR) currently represents one of the most important public health challenges [1]. Any use of antibiotics carries the risk of enhancing the development of AMR [2,3]. The amount of antibiotics used in food-producing animals has recently been below the amount used in human medicine. Nevertheless, it contributes enormously to the overall consumption of antimicrobials (AM) [4]. Both veterinary and human medicine have to address the problem of AMR and promote a more prudent use of antibiotics.

The European Network for Optimization of Veterinary Antimicrobial Treatment (ENOVAT), a COST Action CA 18217, aims to optimize the use of AMs in veterinary medicine, focusing on developing guidelines for AM treatment and improving microbiological diagnostic methods. Members of the project Working Group 4 aim to describe standards for veterinary practice guidelines on the use of AMs, and to establish a priority list of infectious diseases that make up a relatively high proportion of AM consumption in animals [5]. One of them is bovine mastitis. In dairy cows, bovine mastitis represents one of the most common infectious diseases. AM treatment of mastitis remains an important part of mastitis control and represents the main reason for antibiotic treatment in dairy cows [6,7].

To further promote prudent use of antibiotics in Europe in the field of mastitis treatment, it is important to record and understand the current treatment strategies and approaches in the different European countries. On Wednesday, 29 September 2021, a webinar on the topic “Mastitis Treatment in Lactation” was held as part of Working Group 4 of the ENOVAT project. Therein, eight mastitis experts from Spain, The Netherlands, Estonia, Ireland, Poland, Finland, Germany, and Italy described how clinical mastitis in lactation is usually treated in their countries. The aim of this study is to evaluate and compare the different therapeutic approaches and to highlight commonalities and differences. It is intended to help improve understanding of different strategies in European countries and provide a benchmark for comparison between countries. It further aims to help European countries to converge by sharing different views, and thus move towards a common high standard of AM stewardship in veterinary medicine. Finally, it may be possible to come up with urgent research questions that need to be targeted by the scientific community in the near future.

## 2. Results and Discussion

Looking at the therapeutic approaches for clinical mastitis in lactation in the different European countries of Spain, The Netherlands, Estonia, Ireland, Poland, Finland, Germany, and Italy, both differences and similarities become clear. An overview of the comparison of the therapy methods can be seen in Table 1. A more detailed description of the contents of the individual countries can be found in Appendix A.

The testing of milk samples is handled quite differently in the evaluated countries. On the one hand, in countries like Finland, Germany, and Spain, milk samples are often used to detect the pathogen and for the preparation of antibiograms. In Finland, polymerase chain reaction (PCR) is the most common method used in milk sample testing, while in Germany, cultural detection is predominant. In Ireland, farmers often take samples of clinical cases but, due to time constraints, freeze them first and have those analyzed later on. The frequency of the use of milk samples often also depends on the preference of the veterinarian, as is the case in Italy, Poland, and Estonia. Basically, however, little attention is paid to this diagnostic device in Estonia and Poland. Interest in on-farm culture is noticeable in almost all countries. This diagnostic tool is already widely used in some countries like Germany or The Netherlands. In other countries, its use is still in the initial stage, but young veterinarians in particular are interested in it being used more readily.

Regarding the legislative framework for the analysis of milk samples in the individual countries, there is a consensus in Spain, Germany, and Italy. In all three countries, milk samples need to be analyzed when critically important antimicrobials (CIAs) are about to be used. In July 2021, sensitivity testing prior to their use became mandatory in Estonia as well. In Ireland, to date, sampling to justify the use of highest priority critically important antimicrobials (HPCIAs) is only recommended. Finnish legislation requires milk samples to be analyzed regularly, whereas there are no legislative requirements in Poland.

Two different AM classification systems were taken into consideration. The WHO CIA list demonstrates the importance of various AM groups to human medicine: important AM, highly important AM, critically important AM (CIA, with prioritization to high priority and highest priority (HPCIA)) [8]. The European Medicines Agency (EMA) categorization classifies the AMs for prudent and responsible use in animals into four different groups: Category A (“Avoid”), Category B (“Restrict), Category C (“Caution”), Category D (“Prudence”) [9].

In Spain, The Netherlands, Italy and Germany, the use of CIAs has decreased in recent years. Due to fewer licensed Category D products, and aided by the force of habit, in Ireland, almost all tubes contain a CIA, and in 2020, 13% of tubes sold contained an HPCIA. However, the development is positive there, as many co-ops stopped stocking HPCIAs. The use of CIAs can be improved in Poland and Estonia because the use of CIAs is very popular there. In Poland, there is a lack of legal regulations, and the use of antibiotics has even increased over the last years. In Estonia, there have nevertheless been positive developments as well, through voluntary engagement on the part of the scientific community. This has increased the use of penicillin and decreased the use of cephalosporins.

The administration of supportive therapy is an established procedure in more or less all countries, with the use of non-steroidal anti-inflammatory drugs (NSAIDs) predominating. It should be noted that the use also often depends on the preference of the veterinarian, e.g., in Germany and Poland. In The Netherlands, there has been an increase in non-evidence-based mastitis therapy. The Dutch government is promoting the use of natural remedies such as phytotherapeutics to further reduce antibiotic usage.

Furthermore, there are differences in the application of treatment plans. The Netherlands has a model role in this respect, as the 1:1 relationship between veterinarians and farmers, enforced by political pressure, results in herd-based health and treatment plans. In Finland, the preparation of treatment plans and an official herd health contract between a farmer and a veterinarian with regularly scheduled herd visits are prerequisites for leaving antibiotics on the farm in advance for certain commonly occurring diseases. There also exist treatment plans in Germany, but only approximately 30% of them are written down [10]. In most cases, treatment instructions are communicated verbally with the farmer. The Estonian farmers are educated by the veterinarians and also administer antibiotics according to their instructions. In Ireland, there are only a few written treatment plans at all. One reason for this may be the fact that antibiotics for one farm may be prescribed by more than one veterinarian.

Systems and procedures for monitoring antibiotic use are being introduced in more and more countries. In The Netherlands, The Netherlands Veterinary Medicines Institute (SDa) monitors antibiotic use at farm level and thus evaluates national trends. In Finland, all treatments given to food-producing animals must be recorded, which is monitored by the Finnish Food Authority. With the introduction of the electronic prescription system “ricetta elettronica” in 2019 and the database “ClassyFarm,” Italy also started monitoring the use of antibiotics. Electronic prescription of medicines was introduced in Ireland in 2022. With the National Veterinary Prescription System (NVPS), they aim to meet certain requirements within the new EU veterinary medicines regulation 2019/6 [11]. In Spain, electronic prescription became mandatory in 2018, with the result that the use of CIAs, in particular, has dropped. There also exists the National Plan Against Resistance to Antibiotics (PRAN) in Spain which established guidelines to monitor the consumption of antibiotics, control antibacterial resistance, and promote preventive measures. Furthermore, there are regulations for the recording of antibiotic use in Germany, but these currently only apply to meat producers [12]. Monitoring and general analysis of treatments and antibiotic use at farm level are missing in Estonia. There are available data on the sales of veterinary medicines based on wholesalers’ reports, but these data are not divided by species.

With the enforcement of the new EU veterinary medicines regulation 2019/6, a veterinary prescription is mandatory when antibiotics are used in all European countries [11]. However, in Poland, farmers can obtain any antibiotic tube from veterinary clinics at any time without paying a veterinarian a visit. In Ireland, more than one veterinarian can prescribe drugs for a farm, which is a controversial issue as not all veterinarians involved know the farm and/or the farmer well.

The contents of this study need to be viewed within the usual limitations of expert opinions [13]. Due to different mindsets and experiences, there may have been differences in the interpretation of various aspects of the therapy by the speakers. Since the information of the presentation often came from personal experiences or from exchanges with other veterinarians working in the cattle sector, it must be taken into account that these could be anecdotal to some extent. Nevertheless, it can be stated that expert opinions are an important addition, alongside scientific papers, to provide evidence, especially in the practical field of mastitis therapy and its consequences. They help to overcome the gap between clinical research and practical clinical medicine. Thus, they complement empirical evidence and make an overall critical contribution in the context of clinical research. Expert opinion should always be based on clinical experience [14,15]. The experts who participated in this webinar and presented treatment approaches from their respective countries were all veterinarians working in the field of bovine medicine. In addition to their experiences, scientific literature from the respective country was used as a source of information when available. This shows that the information presented is based on both clinical experience and scientific work and can therefore be considered meaningful.

In addition, it should be briefly mentioned that webinars are a relevant platform for knowledge exchange, allowing experts from a wide range of countries to participate and share their knowledge [16]. However, since the length of this webinar and thus the number of participating countries was limited, only a selection of representative European countries could be considered in this study. Therefore, expert opinions from other countries (e.g., United Kingdom, France, and Belgium) are missing. Nevertheless, in selecting the participating countries, emphasis was placed on presenting as broad a picture as possible of European treatment practices for clinical mastitis in lactation. Thematically, the subject area addressed in this webinar was the treatment of clinical mastitis in lactation. To achieve a complete representation of the mastitis treatment of European countries, such as treatment practices of subclinical mastitis and dry cow therapy, further webinars or similar are necessary.

The identified differences between the participating countries in how clinical mastitis in lactation is treated in their respective countries give rise to some research questions that need to be targeted by the scientific community in the near future:

- Systemic treatment versus local treatment: Which treatment method is more effective?
- What is the sufficient treatment duration and how can it be determined?
- Do we need critically important antibiotics in mastitis treatment?
- How much diagnostics is really needed?
- Do different initial situations (like pathogens, AMR, etc.) represent a cause for the described differences?

## 3. Materials and Methods

To reach the goal of optimizing the use of AMs in veterinary medicine, members of the ENOVAT project aim to generate an overview of the current state of microbiological diagnostic procedures and veterinary treatment in Europe. To gain an understanding of the current treatment methods for clinical mastitis in lactation in Europe, an international webinar was scheduled. Various mastitis experts from several European countries were contacted via email and asked to participate in the webinar. They were each requested to prepare a 10 min presentation on their treatment approaches for clinical mastitis in lactation. To give some orientation, a list of possible aspects that they could focus on in their presentations was prepared in advance. The list included the following:

1. Who is responsible for making treatment decisions and for treatment administration in general with regards to bovine clinical mastitis/therapy of high cell count cows in lactation?
2. How does the underlying diagnostic for justification of treatment work?
3. What are the legislative requirements for analyzing milk samples?
4. Are there noteworthy differences in the treatment practice of mastitis based on different production systems?
5. How is an antibiotic treatment commonly administered?
6. How long is antibiotic therapy usually given for, and what criteria are used to assess this?
7. Is supportive treatment commonly used? If yes, what kind, and for which indication?
8. Are broad-spectrum antibiotics used in mastitis treatment? If yes, for which indication?
9. Are critically important antibiotics (CIAs) used in mastitis treatment? If yes, for which indication?
10. Have there been any significant changes in the development of mastitis treatment in your country in the last 10 years?
11. Are there any upcoming tendencies that you would expect to change mastitis treatment practices in your country in the near future?

Eight mastitis experts from Spain, The Netherlands, Estonia, Ireland, Poland, Finland, Germany, and Italy participated in the webinar, all of whom are veterinarians active in a wide variety of fields related to cattle health (Table 2). The speakers were free to choose whether to address the topics of all the points on the orientation list in their presentation, or to pick out a few points that they felt were particularly relevant. Around 200 listeners followed the webinar and actively participated via chat. The webinar took place within the framework of a virtual mobility grant from the ENOVAT project to strengthen existing networks, share knowledge, and learn new techniques. It was held online via a platform of Copenhagen University, Denmark and was recorded for subsequent editing.

## 4. Conclusions

The treatment of intramammary infections represents the main reason for the use of antibiotics in dairy cows. That is why changes are needed in the way clinical mastitis in lactation is treated, not only in individual countries, but across all European countries. To this end, it is necessary to exchange information, views, and approaches, and to promote cooperation at both national and international levels.

There are promising trends in many of the participating European countries. The use of CIAs is already declining in many countries due to pressure from the authorities. In some countries, such as Spain, Germany, Italy, and Estonia, testing of milk samples is mandatory if critical antibiotics are to be used. As already mentioned above, antibiotic use is directly related to the development of AMR [2,3]. That is why the recording and monitoring of antibiotic use is crucial. Fortunately, systems and procedures for recording are being introduced in more and more countries.

In the area of lactational mastitis treatment, increased diagnostic efforts will be necessary in the future to be able to use antibiotics in a more targeted manner. One possible way is the use of rapid on-farm tests, which can often avoid unnecessary use of antibiotics. In addition, a change in drug selection towards narrow-spectrum antibiotics is an important lever for future improvements in antibiotic use. To implement and establish improvement measures, legislative requirements are crucial. Many of the participating countries already have strict legal requirements at the national level that restrict, for example, the use of CIAs. For further Europe-wide improvement of antibiotic use, legal regulations in all European countries are essential. An important contribution has only recently been made at the European level by regulation (EU) 2019/6, which created further legal framework conditions for the best possible use of antibiotics in livestock farming. However, in addition to legal requirements, the willingness of all stakeholders to cooperate, both between countries and at the national level, is key to implementing change. Cooperation between countries allows for the exchange of ideas and learning from each other’s successes and failures. At the farm level, close collaboration between veterinarians and farmers promotes understanding of the benefits of new requirements and ensures their conscientious implementation. For this reason, collaboration at all levels must be promoted in the future, for example, through more webinars like this one.

In the end, it became clear that overall, European countries have a common basis to jointly address the problem of AMR. And since this webinar had about 200 listeners, with some even attending from countries outside Europe, it is clear that the subjects of antibiotics in dairy farming and the related development of resistance in bacteria are topics of global interest.

## Figures and Tables

**Table 1:** Comparison of selected aspects from eight European countries concerning the treatment of clinical bovine mastitis in lactation.

1a. Treatment Decision	Mostly Farm Personnel	Veterinarian	Veterinarian and Farm Personnel	
	ESP_1_, IRL_2_	NLD_3_, FIN_4_, EST_5_, GER_6_	POL_7_ ITA_8_	
1b. Treatment administration	Mostly farm personnel	Veterinarian and farm personnel		
	ESP, NLD, IRL, GER, ITA	EST, POL, FIN		
2a. Diagnostic	All: Clinical symptoms, elevated cell count, etc.			
2b. Bacteriological analysis	Often	Sometimes	Rarely	Not specified
	FIN, GER, ESP	IRL, ITA	EST, POL	NLD
3. Legislative requirements for analysis of milk samples	Existing	Not existing	Not specified	
	For CIAs_9_: ESP, GER, ITA, EST Regularly: FIN	POL, IRL_10_	NLD	
5. Way of treatment	Mostly parenterally	Mostly intramammarily	Equally often	Others
	ESP	NLD, IRL, POL, GER, ITA	EST	FIN_11_
6. Treatment duration	Following label instructions_12_	Extended	Others	
	ESP, IRL, POL	ITA (3–5d imm) EST (4d ± 2d; up to 7–10d) NLD (circa 4d) ESP, POL, FIN, GER (*Staph. aureus*, *Strep. uberis*)	FIN (depends on pathogen) GER (depends on treatment protocol)	
7. Supportive treatment	NSAIDs_13_ used commonly	Other supportive treatment		
	All	NLD (natural remedies)		
9. Use of CIAs	High use	Medium use	Low use	
	POL (esp. fluoroquinolones) EST_14_ (esp. marbofloxacin) ITA_14_, ESP_14_	GER, IRL	FIN, NLD	

_1_ Spain, _2_ Ireland, _3_ The Netherlands, _4_ Finland, _5_ Estonia, _6_ Germany, _7_ Poland, _8_ Italy, _9_ critically important antimicrobials, _10_ guidelines for highest priority critically important antimicrobials (HPCIAs) existent, _11_ depends on the pathogen, _12_ 2–3d intramammary (imm), 3–5d (parenteral), _13_ non-steroidal anti-inflammatory drugs, _14_ drop in the recent time.

**Table 2:** The eight speakers of the webinar “Mastitis Treatment in Lactation”.

Mastitis Expert	Country	Field of Expertise
Demetrio Herrera	Spain	Cofounder of international company Q-Llet
Christian Scherpenzeel	The Netherlands	Dairy veterinary specialist at Royal GD Animal Health
Piret Kalmus	Estonia	Associate Professor at Estonian University of Life Sciences, Institute of Veterinary Medicine and Animal Sciences
Finola McCoy	Ireland	CellCheck Program Manager
Sebastian Smulski	Poland	Veterinary laboratory manager, Assistant Professor at Poznan University of Life Sciences
Päivi Rajala-Schultz	Finland	Professor of Milk Hygiene and Cattle Health at University of Helsinki
Anne Schmenger	Germany	Postdoc at Hannover University of Applied Sciences and Arts
Paolo Moroni	Italy	Professor at University of Milan at the Department of Veterinary Medicine; Director of Quality Milk Production Services Program (QMPS)

## Data Availability

Not applicable.

## Appendix A

### Treatment Methods for Clinical Mastitis in Lactation

Spain: In Spain, treatment decisions are mostly made by farm personnel. They decide which cow needs to be treated, but the veterinarian decides which antibiotic to use. On large dairy farms that employ their own veterinarian, however, the veterinarian decides both. Clinical mastitis cases are often cultured by farmers to detect which group of bacteria is present on the dairy farm and for generating antibiograms.

There has been a change in the way antibiotics are administered in recent years in Spain. Only 10 years ago, mastitis was mainly treated intramammarily. In 2021, however, 55% of all cases were treated parenterally, 40% intramammarily, and in 5% of all cases, those two routes were combined. The duration of treatment is based on the label instructions, so intramammary treatments usually last 2–3 days and parenteral treatments 3–5 days. In some cases in which *Strep. uberis* or *Staph. aureus* is involved and the cure rate is low, an extended therapy of up to 5 days is recommended. However, a longer period is very uncommon. Antibiotic treatment is often supported by the administration of non-steroidal anti-inflammatory drugs (NSAIDs) (especially ketoprofen or carprofen). Here, a new trend can be seen, as some farmers who use on-farm culture tend to use NSAIDs as their first treatment choice when the major group of bacteria is Gram-negative. If clinical symptoms persist after 2–3 days, antibiotic treatment is administered. With this approach, only 20–30% of mastitis cases are treated with AMs without observing a worsening of udder health. However, the implementation of on-farm culture is quite slow, as it involves a considerable increase in effort for the farmers.

As far as critical antibiotics are concerned, a promising change has occurred. In the last decade, Category B antibiotics, like fluoroquinolones and third- and fourth-generation cephalosporins, were the first-choice treatment on many dairy farms. Due to pressure from the authorities and scientists with regards to AMR, a dramatic drop in the application of these treatments is visible. Electronic prescriptions have become mandatory, making it easier to track critically important antibiotic treatments. The National Plan Against Resistance to Antibiotics (PRAN) aims to raise awareness of the imprudent use of antibiotics and has established major guidelines for their usage. In consequence, nowadays Category B antibiotics are only used for hyperacute mastitis caused by Gram-negative bacteria such as *Escherichia coli* or Klebsiella subspecies, and only when diagnostic and antibiograms are performed. The most commonly used antibiotics today are penicillins and sulfonamides.

*The Netherlands*: In The Netherlands, a one-to-one relationship between the veterinarian and the dairy farmer is implemented. Politically enforced results of this relationship have to be farm-specific health and treatment plans. The veterinarian is responsible for making treatment decisions, whereas the farmer and/or the farm personnel carry out the administration based on the treatment plan. This approach has led to an improvement in dairy cow health and animal health in general. An official list created by the Veterinary Antimicrobial Policy Working Group (WVAB) of the Royal Dutch Veterinary Association (KNMvD) details prescribable medicine for dairy cows for a specific indication, and classifies veterinary AMs into first, second, and third choice for use in the existing veterinary treatment guidelines. This dairy formulary represents a guideline for AM use, and with the right veterinary practice directs the focus at deciding which cases of mastitis are worth treating and which are not.

AMs are commonly administered intramammarily (about 70%) in The Netherlands only for proven bacterial intramammary infections, since the Dutch government banned the preventive use of all AMs in animals [17]. The average duration of treatment is, depending on the label, 4 days, and supportive therapy is also common practice. In parallel with the NSAIDs, however, an increase in non-evidence-based mastitis treatments, like the use of natural remedies such as phytotherapeutics, can be observed. The Dutch government aims to spread knowledge of natural remedies and good animal management to support animal health [18].

In the last 10–15 years, political pressure to reduce AM use has increased in The Netherlands. Both farmers and veterinarians are obliged to contribute to a substantial reduction in antibiotic usage. This includes the obligation to make the use of antibiotics transparent using the annual animal-defined daily dose per herd (ADDD). This provides a benchmark for comparing different farms within a country based on AM prescription numbers. The Netherlands Veterinary Medicines Institute (SDa) sets the benchmarks, and monitors and evaluates the national trends to identify problem herds. This process of comparison and benchmarking motivates both farmers and veterinarians to further reduce antibiotic use. Furthermore, a restriction on the use of CIAs was introduced in 2011 and, consequently, the use was limited enormously [19]. In addition, the preventive use of AMs was banned, which especially impacts dry cow therapy and has led to an increase in selective dry cow therapy since 2014. All these requirements resulted in a considerable decrease in antibiotic usage, up to 64%, in the dairy industry in The Netherlands, including CIAs, without deterioration of udder health. Recent years have seen an increase in preventive thinking. Both farmers and veterinarians have become more aware of the selection criteria for cows to be treated during lactation and in the dry period. Therefore, diagnostic testing in a veterinary practice or laboratory (and to some extent on-farm) has been expanded.

*Estonia*: In Estonia, the number of dairy cows was about 85,000, with an average herd size of 140 dairy cows. There are two types of veterinary services. Private practitioners provide their service to several dairy farms, whereas farm-specific veterinarians only work for one dairy farm or company. Antibiotics can only be prescribed by veterinarians. In acute mastitis cases, the farm-specific veterinarians do parenteral treatment themselves, while private practitioners usually use veterinary technicians or skilled farm personnel for parenteral treatments. In both cases, intramammary treatments are normally administered by the milkers and/or the farmers following guidelines by the veterinarian. The ratio of intramammary and parenteral therapy is more or less in proportion, with roughly 50% being treated parenterally. Extended therapy is a common practice in Estonia. The average treatment duration is 4 ± 2 days. In about 15% of the cases, treatment is extended to 7–10 days. Supportive treatment with NSAIDs is frequently used in moderate and severe cases, with ketoprofen as the most common remedy. Currently, available data on the sales of veterinary medicines in Estonia are based on wholesalers` reports collected by State Agency of Medicines and include total sales of veterinary medicines to veterinarians and veterinary pharmacies. However, this information is not itemized by animal species.

Instructions for prudent use of AMs and treatment guidelines were already available in 2012. In July 2021, the use of third- and fourth-generation cephalosporins and fluoroquinolones was restricted in Estonia. If the pathogens are resistant to other AMs, a sensitivity testing became mandatory prior to their use [20]. However, monitoring, general verification, and analysis of the treatments and antibiotic use at farm level are missing. There is also no regulation by the authorities for the milk sample analysis prior to mastitis treatment. Although there are laboratories for testing, and on-farm culture takes place on farms, presumably less than 10% of clinical mastitis cases are analyzed every year in different laboratories.

From 2016–2020, there was a “penicillin promotion campaign.” Together with voluntary national guidelines published in 2017, the aim was to change the attitude and knowledge of both veterinarians and farmers. During this period, educational courses for veterinarians and farmers were organized and many papers were published. As a result, sales of penicillin, especially for intramammary usage, have increased over the years, while those of cephalosporins decreased. However, it is noteworthy that the amount of CIA marbofloxacin has also increased, and thus represents the second most commonly used agent for parenteral treatment, especially for acute cases.

In Estonia, there exists good cooperation with the government, but as there is no restriction at governmental level, voluntary involvement is required. That is why university researchers are trying at an educational level to engage in discourse with both farmers and veterinarians, studying attitudes and behaviors with regards to treatment by participatory epidemiology. They hope that in the near future institutions or the industry itself will start to control the usage of CIAs, and a general database for antibiotic use will be developed.

*Ireland*: In Ireland, antibiotics can be prescribed from the “attending” veterinarian for a bona fide client, but at the same time also from a “co-op vet.” These are veterinarians employed by the dairy co-op. If milk suppliers have signed up to a co-op Mastitis Control Program, they are entitled to apply for their prescription. A prescription is valid for 12 months (this changed with regulation (EU) 2019/6 which came into force in January 2022), and thus, the prescription from the co-op vet is often based on a forecast of the amount of antibiotics the farmer may need. Thus, the farmer often has intramammary tubes—the primary form of administration in Ireland—in stock for administration when needed. Antibiotic treatment is increasingly supported by the administration of NSAIDs. Severe cases are mainly attended to and treated by the veterinarian. Written treatment plans rarely exist, so treatment decisions are very often guided by experience rather than veterinary input or bacteriological culture. The duration of treatment is 3 days on average. Extended therapy sometimes occurs and if so, generally in consultation with the veterinarian who prescribed the agents.

Ireland has a seasonal calving system with over 70% of calving taking place between February and April. This is a labor-intensive time for farmers, so the practicality of sending samples from clinical cases immediately to the laboratory is difficult. Nevertheless, farmers often take samples from clinical cases and freeze them for later analysis. The intention of this is not usually to influence the decision-making for an individual cow, but rather to extend knowledge of the pathogens present on the farm. They also take samples of chronic cases to decide whether treatment is appropriate or not. The average milk recording participation is about 50% of the herds.

Regarding the availability of antibiotic products, it is noticeable that there are few licensed products in Category D. Due to this circumstance, and aided by the force of habit, the use of Category C products is high. There are also highest priority critically important antibiotics (HPCIAs: third-, fourth-, and fifth-generation cephalosporins, glycopeptides, macrolides, and ketolides, polymyxins, quinolones, [8]) licensed for both lactation and dry cow therapy. In recent years, almost all in-lactation tubes sold in Ireland have contained a CIA from Category C, and in 2020, 13% of tubes sold contained an HPCIA, an increase of 7% compared to 2019 [21]. It is also worth noting that many dairy co-ops have stopped stocking HPCIAs since 2019. The quantity of intramammary antibiotics being used, expressed as defined course dose for animals (DCDvet), has decreased since 2012 [21]. One reason for this was the introduction of the national mastitis control program “CellCheck” in 2011.

As an upcoming tendency, there will be changes in the area of dry cow therapy with the regulation (EU) 2019/6 [11]. At the moment, there is a high rate of blanket dry cow therapy. It would also be a good opportunity to redefine who is authorized to prescribe antibiotics and what the required thresholds of knowledge for prescribing should be. For this purpose, the introduction of herd-specific treatment plans would also be beneficial. Further trends are the implementation of an electronic National Veterinary Prescribing System during this year, and an increased availability of licensed narrow-spectrum products for treating bovine mastitis.

*Poland*: Polish dairy herds are relatively small, with an average herd size of less than 20 cows. The stables are often old, manure is usually removed only once a day, and the straw, as bedding material as well as the feed, can be of poor quality. In addition, the milking hygiene is often inadequate. These are the main reasons for bacterial infections of the udder, especially with environmental pathogens.

General treatment decisions are made by veterinarians who are also responsible for the first administration, especially in acute mastitis cases. The following treatments are determined by farmers, zootechnicians or other farm personnel. However, farmers can obtain any antibiotic tube from veterinary clinics at any time without a veterinarian paying them a visit. Antibiotics are commonly administered intramammarily. For specific cases in which *Staph. aureus* or *Streptococcus agalactiae* are involved, parenteral treatments are also used. The average duration of treatment is 2–3 days, with an extended therapy of 5–7 days in cases in which *Strep. uberis* or *Staph. aureus* is the causative pathogen. In acute cases, supportive treatment with antioxidants, selenium, and immunomodulators is given. The use of NSAIDs depends on the attending veterinarian.

Legislative requirements are generally missing in Poland. That is why each veterinarian makes treatment decisions at his or her discretion and without a set procedure. This also influences the usage of antibiotics, which has increased over the years. The use of CIAs is not restricted and, consequently, both quinolones and third- and fourth-generation cephalosporins are quite popular. The analysis of milk samples is not obligatory, so few farmers use this diagnostic tool. All these factors seem to culminate in high AMR rates in Poland.

However, during the last few years, some improvements have been made. Young farmers, in particular, are becoming more interested in the prevention of mastitis as well as in bacteriological examinations. Changing the mentality of both farmers and veterinarians towards putting prophylaxis of mastitis in the foreground, and thus reducing the use of antibiotics in the long term, is viewed as a crucial point.

*Finland (and other Nordic countries)*: A network of mastitis researchers in the Nordic countries of Denmark, Finland, Norway, and Sweden, has jointly developed Nordic Guidelines for Mastitis Therapy (https://www.sva.se/media/qsljw2yb/nordic-guidelines-for-mastitis-therapy.pdf, accessed on 15 October 2021). Practices and legislation differ to some degree between those countries, but the general approach is similar [22].

In Finland, farmers do not have access to veterinary drugs, such as antibiotics, without a veterinary prescription. Farmers detect sick cows and call a veterinarian if required, and veterinarians make treatment decisions and initiate the treatments. Veterinarians can leave drugs on the farm so that farmers can continue the treatment of a specific animal according to veterinary advice. Most farms belong to the national herd health program, and, in that context, a veterinarian and a farmer can make an official contract, whereby the veterinarian visits the farm at regular intervals. In such cases, the veterinarian can leave antibiotics on the farm in advance for the treatment of commonly occurring diseases such as mastitis. This, however, requires detailed written instructions, careful electronic record keeping on the farm, and regular monitoring by the veterinarian that the instructions are followed. Only the amount of medicines that is expected to be needed in between two herd visits can be left on the farm. All veterinarians and farmers are required to keep records of all drugs they administer to food-producing animals.

Initiation of antibiotic treatment for intramammary infections is expected to be based on a microbiologic diagnosis. Milk samples are taken frequently to ensure the most appropriate and targeted treatment, but also to find out whether antibiotics could be omitted. Legislation requires regular bacteriologic analysis, so that etiologic agents of intramammary infections and their AMR profiles in the herd are known, even if not all mastitis cases were sampled. Which antibiotic is used depends on the type of pathogen causing the disease (Gram-positive, Gram-negative, β-lactamase−, β-lactamase+), but in general, penicillin is the drug of choice. As far as CIAs are concerned, it can be noted that no cephalosporin products are licensed and on the market for intramammary treatments. Fluoroquinolones are licensed for parenteral treatment of severe infections caused by Gram-negative bacteria. For such intramammary infections cases, however, the first choice for treatment is no antibiotics, but only supportive therapy. Recommendations for the route of administration and length of antibiotic treatments of intramammary infections depend on the etiologic agent and vary between the Nordic countries. In Finland, a 3-day intramammary treatment for mild and moderate clinical cases caused by most Gram-positive bacteria is used. Treatments of *Staph. aureus* and *Strep. uberis* intramammary infections are extended to 5 days, often using a combination of parenteral and intramammary therapy. Supportive treatment, such as fluid therapy or anti-inflammatories, is used in all severe cases.

Although the Nordic countries already have followed the prudent use of antibiotics guidelines, and thus have low antibiotic consumption rates, there is still interest in further reduction by focusing on prevention [22].

*Germany*: Regarding antibiotic therapy, a clinical examination of the sick animal by the veterinarian is required [23]. Antibiotics are then prescribed by the veterinarian for that specific animal. A veterinarian may dispense prescription drugs for food-producing animals in an amount intended for use within the following 31 days. Medicinal products containing AM agents that are not exclusively applied locally may only be dispensed for the following 7 days [12]. The administration of treatment is mostly done by milkers or the herdsman [10]. Normally, farmers have a few udder tubes on the farm to start treatment according to treatment instructions made by the veterinarian. Accordingly, antibiotics are mostly administered intramammarily. Additional systemic AM treatment depends on the veterinarian and is mostly given in severe cases. Many farmers finish therapy after completing the instructions on the treatment protocol for mastitis, others stop when flakes are no longer detected in the milk [10]. If clinical signs persist, or *Strep. uberis* or *Staph. aureus* is detected, therapy is sometimes extended. Supportive treatment, such as the use of NSAIDs, is used commonly but always depends on the preference of the treating veterinarian.

Although farmers are not legally obliged to have milk samples bacteriologically tested, about 70% of farmers do so regularly. The most common methods here are cultural detection and resistance tests. When it comes to the use of CIAs, a resistance test has been mandatory since the Amendment of Veterinary Pharmacy Regulation in 2018 [23]. As a result, many veterinarians stopped using CIAs and farmers started to realize that it is important to use fewer CIAs in the future. In a survey conducted in 2019, only 17% of the farms mentioned using CIAs at all, and then, only used them in severe cases [24].

Notably, a targeted therapy concept, serving as a guideline for more evidence-based therapy decisions, is gradually being adopted, especially in northern and central Germany. It can be used by both farmers and veterinarians. This procedure is based on an on-farm test in combination with the identification of animals worthy of treatment. Using this therapy concept, 73% of intramammary antibiotic doses and 65% of systemic antibiotics could be reduced [25].

*Italy*: In Italy, there have recently been major changes. Monitoring of the use of antibiotics started with the introduction of an electronic prescription (“ricetta elettronica”) in 2019. With this implementation, and the fact that veterinarians in Italy do not sell drugs, farmers can buy all medicines indicated in the veterinary prescription from an external pharmacy. The treatment decision thus lies with the veterinarian today, whereas until 2019, the situation was quite similar to Spain, with mostly farmers and/or milkers making treatment decisions. The milkers then initiate the treatments based on veterinary protocols. Furthermore, “ClassyFarm,” a database collecting drug prescriptions, animal welfare, and production data from Italian farms, was introduced, which allows the analysis of antibiotic use for each farm.

Antibiotics are generally administered intramammarily based on label recommendations. Supportive treatment with anti-inflammatories has started to be part of the protocol in most clinical mastitis cases, also presenting a major change. In Italy, a specific length of treatment based on the etiology of the mastitis case does not exist, except for the herds where on-farm culture is performed. Important factors in this context are the level of experience of the veterinarian as well as the label instructions on the product. In some cases, therapy is extended for 5–7 days, with an increase in antibiotic withdrawal times. The veterinarian is also the decision-maker when it comes to cultural testing of milk samples. On-farm tests are only used on about 6% of farms, usually on large ones that are supervised by young veterinarians interested in milk quality. Other veterinarians collect clinical mastitis cases every month and send them to the laboratory to detect any change in epidemiology and to carry out resistance tests. Conversely, however, there are also farms performing no culture tests at all. Nonetheless, when a CIA is about to be used, legislation requires cultural analysis and resistance tests. In 2004, cephalosporins were the first choice of treatment, followed by fluoroquinolones [26]. Today, influenced by the implementation of electronic prescriptions, the use of third- and fourth-generation cephalosporins has decreased, and first-generation cephalosporins and penicillin are experiencing more widespread application.

As an upcoming tendency, there is a discussion whether milk payment will not only be based on yield, butter fat, protein, and SCC, but also on the basis of welfare and reduction in the use of antibiotics. Furthermore, sharing information and the promotion of collaborations, both at a national level between different institutions and at an international level, is viewed as an important task to aid udder health.
